# Targeting HER2-Positive HCC1954 Breast Cancer Cells by Novel Thiazole-Dihydrobenzisoxazoles: In-Depth Design, Synthesis and Initial *In Vitro* Study

**DOI:** 10.32604/or.2025.067832

**Published:** 2025-11-27

**Authors:** Yuri A. Piven, Danila V. Sorokin, Nastassia A. Varabyeva, Alexandra L. Mikhaylova, Fedor B. Bogdanov, Elena V. Shafranovskaya, Raman M. Puzanau, Fedor A. Lakhvich, Alexander M. Scherbakov

**Affiliations:** 1 Institute of Bioorganic Chemistry, National Academy of Sciences of Belarus, Minsk, 220084, Belarus; 2 Department of Experimental Tumor Biology, Blokhin N.N. National Medical Research Center of Oncology, Moscow, 115522, Russia; 3 Health Institution “National Anti-Doping Laboratory”, Minsk, 223040, Belarus; 4 Gause Institute of New Antibiotics, Moscow, 119021, Russia

**Keywords:** Anticancer therapy, breast cancer, heat shock protein 90 (HSP90), human epidermal growth factor receptor 2 (HER2), epidermal growth factor receptor (EGFR), dual inhibitors

## Abstract

**Background:**

The most aggressive forms of breast cancer are characterized by independence from steroid hormones but a strong dependence on growth factors. In such cancer cells, oncogenic receptors, including human epidermal growth factor receptor 2 (HER2), are activated, and their targeted inhibition represents an attractive therapeutic strategy. The study aimed to develop small-molecule potential dual heat shock protein 90 (HSP90)-HER2 inhibitors and evaluate them as anticancer agents in HER2-positive cells.

**Methods:**

The research project involved obtaining a series of compounds with potential dual inhibitory activity against HSP90 and HER2 by targeted organic synthesis, which was preliminarily assessed using molecular modelling and calculation of key parameters of molecular dynamics. The potential therapeutic benefit of the obtained molecules was studied using basic molecular biological methods, including assessment of cytotoxic activity *in vitro* using the MTT test, as well as determination of a possible mechanism of action based on the expression of key participants in intracellular signaling (western blotting). Additionally, therapeutic combinations were developed and tested on a cellular model of the disease, including a lead compound and chemotherapeutic drugs used in clinical practice, in order to find synergistic pairs and improve the effectiveness of the treatment.

**Results:**

In this work, novel dual HSP90-HER2 inhibitors, based on the fused thiazole-dihydrobenzisoxazole polycyclic scaffold, were designed and synthesized. The resulting compounds exhibited strong antiproliferative activity against HER2-positive breast cancer cells with high selectivity. Among them, **ATF-2** demonstrated antiproliferative activity comparable to HER2 inhibitor lapatinib and significantly suppressed HER2 expression and activity, epidermal growth factor receptor (EGFR) activity, and cyclin-dependent kinase 6 (CDK6) expression in HCC1954 breast cancer cells.

**Conclusion:**

These findings highlight **ATF-2** as a promising dual HSP90-HER2 inhibitor with broader inhibitory effects on the HER2, EGFR, and CDK6 pathways.

## 1 Introduction

According to recent World Health Organization data, more than 19 million new cancer cases are diagnosed each year, with annual cancer-related deaths nearing 10 million. Although cancer predominantly affects older populations, a growing number of younger individuals are being diagnosed [1]. This shift is influenced by several factors, including exposure to harmful substances, poor lifestyle habits, inherited genetic mutations, and environmental pollutants [2]. In low-income countries, limited access to quality medical care and insufficient early diagnosis worsen outcomes [3]. Conversely, in wealthier nations, cancer incidence is rising due to the aging population and lifestyle-related risks such as unbalanced diets and physical inactivity [4].

Breast cancer stands as the most frequently diagnosed malignancy in women and is among the top three most common cancers globally, along with lung and colorectal cancers [5]. In 2022 alone, it affected approximately 2.3 million women and caused around 670,000 deaths [6]. Breast cancer refers to a group of malignant tumors that typically originate in the terminal ductal lobular units of the breast [7]. Each tumor type is characterized by distinct molecular and histological features, which are shaped by both genetic and epigenetic changes [8]. A key molecular marker in many cases is human epidermal growth factor receptor 2 (HER2), a membrane receptor that facilitates cell growth and division. Unlike other members of the ErbB receptor family, HER2 does not bind ligands directly but instead acts as a preferred partner in receptor dimerization [9]. Amplification of the ERBB2 gene on chromosome 17q12 [10] leads to HER2 overexpression in 15%–25% of breast cancers, driving aggressive tumor behavior and poor clinical outcomes [11]. While HER2 serves as an established therapeutic target and a predictive biomarker, particularly in HER2-positive gastric cancers, its prognostic value remains under debate [12,13]. Because HER2 plays a central role in tumor development, it continues to be a primary focus of drug development in oncology.

Targeted therapies against HER2 began with the introduction of monoclonal antibodies (mAbs). While these antibodies alone have limited tumor-killing ability, they tend to be well tolerated. Trastuzumab, the first humanized anti-HER2 mAb, blocks HER2-mediated signaling and activates antibody-dependent cell-mediated cytotoxicity (ADCC) in HER2-overexpressing tumors [14]. Combining trastuzumab with chemotherapy (cisplatin plus either capecitabine or 5-fluorouracil) remains the standard first-line treatment for advanced HER2-positive breast and gastric cancers [15]. Pertuzumab is another mAb used to treat HER2-positive cancers; it binds to domain II of the receptor’s extracellular region, preventing dimerization with other ErbB proteins and interrupting signaling. A newer agent, margetuximab, targets the same HER2 epitope as trastuzumab but is engineered to enhance immune response via ADCC [16]. Findings suggest that margetuximab may offer comparable efficacy to trastuzumab-based combinations, even as a standalone therapy [17–19].

Beyond antibodies, small-molecule tyrosine kinase inhibitors (TKIs) also play a vital role in HER2-targeted therapy. Lapatinib, an oral agent derived from 4-anilinoquinazoline, selectively inhibits HER2/ErbB2 and epidermal growth factor receptor (EGFR)/ErbB1 by reversibly blocking the ATP-binding pocket of their intracellular kinase domains [20]. Like other TKIs such as erlotinib and gefitinib, lapatinib competes with ATP to disrupt signaling, slowing tumor cell growth and triggering apoptosis [21]. Laboratory studies confirm its effectiveness against various HER2- and EGFR-expressing cancer cells, including those from breast, lung, and gastric origins [22]. Clinically, lapatinib is effective both alone and in combination with trastuzumab, even in patients who have developed resistance to trastuzumab. Nevertheless, the emergence of mutations—such as L755S, L755P, and T798M—can limit its effectiveness [23,24], highlighting the need for next-generation agents with better resistance profiles [25].

One of the most promising advancements in HER2-targeted treatments is the rise of antibody–drug conjugates (ADCs). Following trastuzumab’s success, researchers developed conjugates that combine targeted delivery with potent cytotoxic drugs. The first HER2-specific ADC, trastuzumab emtansine (T-DM1), was approved in 2013 for treating advanced HER2-positive breast cancer [26]. T-DM1 links trastuzumab with DM1, a microtubule-disrupting agent, using a stable non-cleavable linker [27]. More recently, trastuzumab deruxtecan (T-DXd) has shown transformative potential. This ADC connects trastuzumab to deruxtecan—a strong topoisomerase I inhibitor—via a linker that is cleaved by cathepsin enzymes [28]. Both experimental [29] and clinical studies confirm T-DXd’s efficacy in HER2-positive, trastuzumab-resistant breast cancers, as well as HER2-mutant non-small-cell lung cancer [30–32].

Despite these strides, ADC development faces challenges such as regulatory complexity and high production expenses. An alternative strategy involves creating dual inhibitors that simultaneously disrupt multiple cancer-related pathways. One such target is heat shock protein 90 (HSP90), a molecular chaperone involved in stabilizing, folding, and degrading client proteins [33–35]. HSP90 exists in several cellular compartments: HSP90α and HSP90β in the cytoplasm [36], GRP94 in the endoplasmic reticulum [37], and TRAP-1 in mitochondria [38]. These proteins are integral to processes such as stress response, apoptosis, and cell division [39]. Given that many of HSP90’s client proteins are implicated in cancer, targeting HSP90 is a compelling therapeutic approach [40]. Luminespib (NVP-AUY922), a potent isoxazole-based HSP90 inhibitor with an IC_50_ of 21 nM, has shown antitumor activity in preclinical models [41]. However, clinical trials have revealed limited success due to side effects, including vision-related toxicity [42].

## 2 Materials and Methods

### 2.1 Molecular Modeling

#### 2.1.1 Molecular Docking

Five conformers of the designed potential HSP90-HER2 dual inhibitors **ATF-1** and **ATF-2** were prepared with DataWarrior software v. 06.04.02 (Actelion, Allschwil, Switzerland) [43] using MMFF94s+ forcefield. The sdf file was converted into separate mol2 files using Open Babel v. 3.1.0 [44]. The script “prepare_ligand4.py” from the ‘AutoDockTools’ package v. 1.5.7 (Scripps Research, San Diego, CA, USA) [45] was used for converting mol2 files into pdbqt files. The ligands and water were removed from the receptor proteins (PDB ID 6LTI for HSP90α and PDB ID 3PP0 for HER2) for the protein preparations. Missing internal fragments of the HER2 protein were modelled in ChimeraX v. 1.9 (University of California San Francisco, San Francisco, CA, USA) [46] using MODELLER plugin [47]. ‘AutoDockTools’ was used for the conversion of protein PDB files into pdbqt files. The following parameters of grid boxes were defined:

HSP90 (6LTI): ΔX = ΔY = ΔZ = 24 Å centered at X = 34 Å, Y = −9 Å, Z = −25 Å.

HER2 (3PP0): ΔX = ΔY = ΔZ = 24 Å centered at X = 16 Å, Y = 17 Å, Z = 27 Å.

QVina2 software v. 2.1 [48] was used for conducting docking experiments. The predicted poses, which have higher affinity values, were selected for further analysis. Discovery Studio Visualizer v. 21.1 (BIOVIA, Cambridge, UK) and PyMol v. 3.1.0 (Schrödinger, New York, NY, USA) were used for image preparation and visualization.

#### 2.1.2 Molecular Dynamics Simulations and Molecular Mechanics/Generalized Born Surface Area (MM/GBSA) Calculations

The resulting docking complexes underwent molecular dynamics simulations with GROMACS v. 2024.3 [49], using AMBER99SB-ILDN forcefield [50]. ACPYPE [51] was used for ligand parametrization (bond charge correction (BCC) charges and general AMBER Force Field (GAFF)). At the first stage, complexes were dissolved in the water (transferable intermolecular potential 3P (TIP3P)) in a dodecahedron box. The systems were made electrically neutral by adding Na^+^ or Cl^−^ ions. After energy minimization (steepest descent), NVT (constant number, volume, temperature; 1 ns) and NPT (constant number, pressure, temperature; 1 ns) equilibrations, 200 ns or 1000 ns simulations in the NPT ensemble were carried out at constant pressure and temperature (1 bar and 300 K). gmx_MMPBSA software v. 1.6.3 [52] was used to calculate ΔH values (GB-HCT model l [53] and single trajectory approximation). For decomposition analysis on the basis of 1000 ns simulation trajectories, the gmx_MMPBSA software was used.

### 2.2 Chemistry

#### 2.2.1 General Information

Thionyl chloride, triethylamine and dimethyl sulfoxide-d6 (DMSO-d_6_) were purchased from Sigma-Aldrich (Saint Louis, MO, US). 4-Dimethylaminopyridine (DMAP) was purchased from TCI (Tokyo, Japan). The solvents were purchased from local suppliers and distilled before use by standard methods. 5-Bromo-3-(5-isopropyl-2,4-dimethoxyphenyl)-6,7-dihydrobenzo[*d*]isoxazol-4(5*H*)-one **1b** and 8-(5-isopropyl-2,4-dimethoxyphenyl)-4,5-dihydrothiazolo [4,5^′^:3,4]benzo[1,2-*c*]isoxazol-2-amine **2a** were prepared using methods described in [54,55], respectively. ^1^H, ^13^C, and ^19^F NMR spectra were registered on a Bruker AVANCE 500 spectrometer (Bruker, Billerica, MA, USA; 500 MHz for ^1^H, 126 MHz for ^13^C and 470 MHz for ^19^F). Chemical shift values are given in δ (ppm) relative to the residual solvent signals for δ_H_ 2.50 ppm (DMSO-d_6_) and δ_C_ 39.52 ppm (DMSO-d_6_). Constants (J) are given in Hertz (Hz). Multiplicity of signals was indicated as follows: s (singlet), d (doublet), t (triplet), q (quartet), hept (heptet), m (multiplet), br.s (broad singlet), or combinations thereof. High resolution MS was obtained on a Thermo Fisher Scientific Q-Exactive-Plus LC/MS system (Thermo Fisher Scientific, Waltham, MA, USA) using heated electrospray ionization (HESI). An Agilent 1200 RRPL High-Performance Liquid Chromatography (HPLC) instrument (Agilent Technologies, Santa Clara, CA, USA) was used for high-performance liquid chromatography (HPLC). Boetius PHMK 78/1742 apparatus (VEB Analytik, Dresden, Germany) was used for melting point determination. Flash column chromatography was performed through silica gel (70–230 mesh) or neutral aluminum oxide. Pre-coated silica gel or alumina F254 plates were used for Thin-Layer Chromatography (TLC) progress monitoring of the reactions. All compounds are >95% pure by HPLC. NMR data (^1^H, ^13^C, ^19^F), HRMS data, and HPLC data are provided in electronic supplementary information (ESI).

#### 2.2.2 Synthesis and Characterization of Compounds

*Synthesis of* ***2b***:

**8-(5-Isopropyl-2,4-dimethoxyphenyl)-4,5-dihydrothiazolo [5^′^,4^′^:5,6]benzo[1,2- *d*]isoxazol-2-ami ne (2b)**: Synthesized according to the procedure described in the literature [55,56]. Yield 76%. Yellow solid, mp > 210°C (dec.). ^**1**^**H NMR** (500 MHz, DMSO-*d*_6_) δ 7.12 (s, 1H), 6.71 (br. s, 3H), 3.88 (s, 3H), 3.70 (s, 3H), 3.18 (hept, *J* = 6.9 Hz, 1H), 3.14–3.07 (m, 2H), 3.03–2.96 (m, 2H), 1.14 (d, *J* = 6.9 Hz, 6H). ^**13**^**C NMR** (126 MHz, DMSO-*d*_6_) δ 169.0 (C), 167.0 (C), 158.5 (C), 157.1 (C), 155.3 (C), 139.2 (C), 127.7 (CH), 127.2 (C), 111.9 (C), 111.6 (C), 109.6 (C), 96.4 (CH), 55.8 (CH_3_), 55.7 (CH_3_), 25.7 (CH), 22.8 (2CH_3_), 21.7 (CH_2_), 21.1 (CH_2_). **HRMS** (HESI): *m/z* calc’d for C_19_H_22_N_3_O_3_S [M+H]^+^: 372.13764, found 372.13770.

*General procedure A for the synthesis of amides* ***4***:

A round-bottomed flask was charged with 3-(3-fluorobenzamido) benzoic acid **3** (280 mg, 1.08 mmol, 2 eq.) and thionyl chloride (8 mL, 110 mmol, 204 eq.). The resulting mixture was refluxed under an argon atmosphere for 6 h. After completion of the reaction (TLC analysis), thionyl chloride was removed under reduced pressure. The resulting 3-(3-fluorobenzamido)benzoyl chloride was dissolved in dry CHCl_3_ (5.4 mL) followed by addition of amine **2a** or **2b** (200 mg, 0.54 mmol, 1 eq.), triethylamine (376 μL, 2.7 mmol, 5 eq.) and DMAP (6 mg, 0.05 mmol, 0.1 eq.). The reaction mixture was heated under reflux under an argon atmosphere for 24 h. Chloroform and triethylamine were evaporated under reduced pressure. The residue was flash chromatographed on neutral Al_2_O_3_ with CHCl_3_ to get rid of acyl chloride. And the product was additionally purified by column chromatography on silica gel (30 → 35% EtOAc/PE).

**3-Fluoro-N-(3-((8-(5-isopropyl-2,4-dimethoxyphenyl)-4,5-dihydrothiazolo [4^′^,5^′^:3,4]benzo[1,2- *c*] isoxazol-2-yl)carbamoyl)phenyl)benzamide (4a):** Yield 54%. Yellow solid, mp > 240°C (dec.). ^1^H NMR (500 MHz, DMSO-*d*_6_) δ 12.52 (s, 1H), 10.53 (s, 1H), 8.43 (t, *J* = 2.0 Hz, 1H), 8.00 (ddd, *J* = 8.2, 2.1, 0.9 Hz, 1H), 7.87–7.79 (m, 3H), 7.61 (td, *J* = 8.0, 5.8 Hz, 1H), 7.53–7.45 (m, 2H), 7.30 (s, 1H), 6.77 (s, 1H), 3.92 (s, 3H), 3.77 (s, 3H), 3.20 (hept, *J* = 6.9 Hz, 1H), 3.15 (t, *J* = 7.6 Hz, 2H), 3.05 (t, *J* = 6.9 Hz, 2H), 1.16 (d, *J* = 6.9 Hz, 6H). ^13^C NMR (126 MHz, DMSO-*d*_6_) δ 165.0 (C), 164.3 (C), 162.0 (d, *J* = 244.3 Hz, C), 161.5 (C), 159.2 (C), 158.1 (C), 156.9 (C), 156.5 (C), 139.2 (C), 137.9 (C), 136.9 (d, *J* = 7.3 Hz, C), 132.6 (C), 130.7 (d, *J* = 8.0 Hz, CH), 128.8 (CH), 127.8 (C), 127.7 (CH), 124.3 (CH), 124.0 (CH), 123.5 (CH), 123.3 (C), 120.6 (CH), 118.7 (d, *J* = 20.9 Hz, CH), 114.6 (d, *J* = 22.8 Hz, CH), 109.3 (C), 108.3 (C), 96.3 (СΗ), 55.8 (2CH_3_), 25.7 (CH), 22.6 (2CH_3_), 20.8 (CH_2_), 20.5 (CH_2_). ^19^F NMR (470 MHz, DMSO-*d*_6_) δ −112.50–−112.59 (m). HRMS (HESI): *m/z* calc’d for C_33_H_30_FN_4_O_5_S [M+H]^+^: 613.19155, found 613.19067.

**3-Fluoro-N-(3-((8-(5-isopropyl-2,4-dimethoxyphenyl)-4,5-dihydrothiazolo [5^′^,4^′^:5,6]benzo[1,2- *d*]isoxazole-2-yl)carbamoyl)phenyl)benzamide (4b):** Yield 27%. Yellow solid, mp > 220°C (dec.). ^1^H NMR (500 MHz, DMSO-*d*_6_) δ 12.50 (s, 1H), 10.53 (s, 1H), 8.40 (t, *J* = 2.0 Hz, 1H), 8.00 (dd, *J* = 8.3, 2.3 Hz, 1H), 7.86–7.83 (m, 2H), 7.83–7.79 (m, 1H), 7.61 (td, *J* = 8.0, 5.8 Hz, 1H), 7.53–7.45 (m, 2H), 7.20 (s, 1H), 6.78 (s, 1H), 3.91 (s, 3H), 3.70 (s, 3H), 3.25–3.17 (m, 5H), 1.15 (d, *J* = 6.9 Hz, 6H). ^13^C NMR (126 MHz, DMSO*-d*_6_) δ 169.9 (C), 165.0 (C), 164.3 (C), 162.0 (d, *J* = 244.8 Hz, C), 158.7 (C), 157.2 (C), 156.3 (C), 155.3 (C), 139.3 (C), 139.1 (C), 136.9 (d, *J* = 6.5 Hz, С), 132.8 (C), 130.7 (d, *J* = 8.3 Hz, CH), 128.8 (CH), 127.4 (C), 127.3 (CH), 124.2 (CH), 124.0 (CH), 123.4 (CH), 120.5 (CH), 119.4 (С), 118.7 (d, *J* = 21.0 Hz, CH), 114.6 (d, *J* = 23.1 Hz, CH), 111.4 (С), 109.3 (С), 96.5 (CH), 55.7 (CH_3_), 55.7 (CH_3_), 25.7 (CH), 22.7 (2CH_3_), 21.6 (CH_2_), 20.9 (CH_2_). ^19^F NMR (470 MHz, DMSO-d6) δ −112.51–−112.58 (m). HRMS (HESI): *m/z* calc’d for C_33_H_30_FN_4_O_5_S [M+H]^+^: 613.19155, found 613.19061.

*General procedure B for the synthesis of* ***ATF***:

To a dry round-bottomed flask equipped with a stir bar, amide **4** (80 mg, 0.13 mmol, 1 eq.) was added, followed by the addition of dry 1,2-dichloroethane (DCE) (1.3 mL). The flask was allowed to purge under argon, after which BBr_3_ (98 μL, 1.04 mmol, 8 eq.) was added slowly at 0°C. The reaction mixture was stirred at room temperature until the starting material was consumed completely as indicated by TLC (72 h). The reaction was quenched with 1M HCl, and the organic layer was separated from the aqueous layer. The aqueous layer was then extracted with EtOAc. The organic layers were combined and dried over Na_2_SO_4_. After removing the solvent with a rotary evaporator, the crude product was purified by silica gel column chromatography (35 → 40% EtOAc/PE).

**N-(8-(2,4-dihydroxy-5-isopropylphenyl)-4,5-dihydrothiazolo [4^′^,5^′^:3,4]benzo[1,2-*c*] isoxazol-2-yl) -3-(3-fluorobenzamido)benzamide (ATF-1):** Yield 26%. Yellow solid, mp 250°C–255°C. ^1^H NMR (500 MHz, DMSO-*d*_6_) δ 12.65 (s, 1H), 10.53 (s, 1H), 9.73 (s, 1H), 9.61 (s, 1H), 8.45 (t, *J* = 2.0 Hz, 1H), 7.99 (ddd, *J* = 8.4, 2.2, 1.0 Hz, 1H), 7.87–7.79 (m, 3H), 7.61 (td, *J* = 8.1, 5.8 Hz, 1H), 7.53–7.48 (m, 1H), 7.46 (ddd, *J* = 8.3, 2.7, 0.9 Hz, 1H), 7.20 (s, 1H), 6.49 (s, 1H), 3.17–3.06 (m, 3H), 3.03 (t, *J* = 7.6 Hz, 2H), 1.15 (d, *J* = 6.9 Hz, 6H). ^13^C NMR (126 MHz, DMSO-*d*_6_) δ 165.0 (C), 164.3 (C), 162.0 (d, *J* = 244.4 Hz, C), 161.5 (C), 159.3 (C), 157.4 (C), 156.7 (C), 154.6 (C), 139.2 (C), 138.1 (C), 136.9 (d, *J* = 6.3 Hz, C), 132.6 (C), 130.7 (d, *J* = 7.9 Hz, CH), 128.9 (CH), 128.0 (CH), 125.8 (C), 124.4 (CH), 124.0 (CH), 123.3 (CH), 123.2 (C), 120.6 (CH), 118.7 (d, *J* = 21.1 Hz, CH), 114.6 (d, *J* = 22.9 Hz, CH), 108.5 (C), 106.4 (C), 103.4 (CH), 25.9 (CH), 22.6 (2CH_3_), 20.8 (CH_2_), 20.6 (CH_2_). ^19^F NMR (470 MHz, DMSO-*d*_6_) δ −112.48–−112.57 (m). HRMS (HESI): *m/z* calc’d for C_31_H_26_FN_4_O_5_S [M+H]^+^: 585.16025, found 585.15961.

**N-(8-(2,4-dihydroxy-5-isopropylphenyl)-4,5-dihydrothiazolo [5^′^,4^′^:5,6]benzo[1,2- *d*]isoxazol-2-yl)-3-(3-fluorobenzamido)benzamide (ATF-2):** Yield 38%. Yellow solid, mp 230–235°C. ^1^H NMR (500 MHz, DMSO-*d*_6_) δ 12.59 (s, 1H), 10.53 (s, 1H), 9.55 (s, 1H), 9.31 (s, 1H), 8.45 (t, *J* = 2.0 Hz, 1H), 7.97 (ddd, *J* = 8.1, 2.2, 0.9 Hz, 1H), 7.89–7.77 (m, 3H), 7.62 (td, *J* = 8.0, 5.8 Hz, 1H), 7.54–7.43 (m, 2H), 7.22 (s, 1H), 6.47 (s, 1H), 3.25–3.08 (m, 5H), 1.16 (d, *J* = 6.9 Hz, 6H). ^13^C NMR (126 MHz, DMSO-*d*_6_) δ 170.2 (C), 164.9 (C), 164.3 (C), 162.0 (d, *J* = 244.8 Hz, С), 156.8 (C), 156.3 (C), 156.1 (C), 154.8 (C), 139.5 (C), 139.1 (C), 136.9 (d, *J* = 7.2 Hz, С), 132.7 (C), 130.7 (d, *J* = 8.0 Hz, CH), 128.8 (CH), 128.0 (CH), 125.1 (C), 124.3 (CH), 124.0 (CH), 123.4 (CH), 120.5 (CH), 119.6 (C), 118.8 (d, *J* = 21.5 Hz, CH), 114.6 (d, *J* = 23.1 Hz, CH), 111.2 (C), 10,679 (C), 103.1 (CH), 25.9 (CH), 22.7 (2CH_3_), 21.7 (CH_2_), 20.9 (CH_2_). ^19^F NMR (470 MHz, DMSO-*d*_6_) δ −112.45–−112.60 (m). HRMS (HESI): *m/z* calc’d for C_31_H_26_FN_4_O_5_S [M+H]^+^: 585.16025, found 585.15948.

### 2.3 Biology

#### 2.3.1 Cell Culture Conditions, Antiproliferative Assay, and Selectivity Evaluation

The HCC1954 human breast cancer cell line [57] was obtained from the American Type Culture Collection (ATCC, Manassas, VA, USA), while E. Dashinimaev generously supplied the hTERT-immortalized human skin fibroblasts (hFB-hTERT) [58]. Cells were authenticated by morphology and STR profiling by Gordiz (Moscow, Russia, http://gordiz.ru/). Mycoplasma contamination was excluded using DNA staining methods as recommended in [59–61]. Both cell types were cultivated in RPMI 1640 medium (PanEco, Moscow, Russia), enriched with 10% fetal bovine serum (FBS, Hyclone, Thermo Fisher Scientific, Logan, UT, USA) and essential vitamins (PanEco). Cultures were maintained under standard conditions at 37°C in an atmosphere containing 5% CO_2_ and 75%–85% relative humidity.

To assess antiproliferative activity, a modified MTT assay was employed, adapting protocols from previous studies [62,63]. Cells were seeded into 24-well plates (Corning Inc., Corning, NY, USA) at densities of 5 × 10^4^ cells/well for HCC1954 and 6 × 10^4^ cells/well for hFB-hTERT, using 900 μL of medium per well. Test compounds (**ATF-1** and **ATF-2**) were initially dissolved in dimethyl sulfoxide (DMSO, chemically pure (CP), not less than 99.8 wt%) to prepare 5 mM stock solutions and were diluted in culture medium to desired working concentrations immediately prior to application.

After allowing cells to adhere for 24 h, 100 μL of compound-containing medium was added per well, and incubation continued for 72 h. Concentration-dependent responses in the range of 0.1–25 μM were analyzed. At the end of treatment, the medium was replaced with MTT solution (0.2 mg/mL), and after 1 h of incubation, the resulting formazan was solubilized using 350 μL of DMSO per well. Plates were gently agitated to ensure complete dissolution, and absorbance at 571 nm was measured using a MultiSkan microplate reader (Thermo Fisher Scientific, Life Technologies Holdings Pte. Ltd., Singapore). Cell viability percentages were calculated after correcting for background signals. Half-maximal inhibitory concentrations (IC_50_) were derived using nonlinear regression modeling via GraphPad Prism software v. 8.0.1 (GraphPad Software, Boston, MA, USA).

The selectivity index (SI) for each compound was calculated by dividing the IC_50_ in hFB-hTERT cells by the corresponding IC_50_ in HCC1954 cells, offering a quantitative measure of tumor selectivity.

$$SI=\frac{IC_{50}(\text{hFB-hTERT})}{IC_{50}(\text{HCC}1954)}$$

#### 2.3.2 Immunoblotting Procedure

HCC1954 cells were seeded into 100-mm dishes (Corning), and after 24 h, experimental compounds **ATF-1** and **ATF-2** were introduced into fresh medium. Following another 24-h incubation period, cells were harvested for protein extraction. After two washes with ice-cold PBS, cells were lysed on ice for 10 min in buffer containing 50 mM Tris-HCl (pH 7.5), 1% Igepal CA-630, 150 mM NaCl, 1 mM EDTA, 1 mM DTT, 1 mM sodium orthovanadate, 1 mM sodium fluoride, and a protease inhibitor mix (bestatin, leupeptin, and pepstatin, each at 1 μg/mL), as described by Zapevalova et al. [64]. Protein concentration was determined using the Bradford assay (Bio-Rad Protein Assay Dye Reagent Concentrate, Bio-Rad, Hercules, CA, USA) [65].

Proteins were resolved by SDS-PAGE using 10% gels under reducing conditions and transferred onto nitrocellulose membranes (GE Healthcare, Chicago, IL, USA). Membranes were blocked in TBS containing 5% non-fat dry milk and 0.1% Tween-20 to prevent nonspecific antibody binding. Primary antibodies were incubated overnight at 4°C.

Detection was carried out using antibodies targeting both phosphorylated and total forms of HER2 (cat. # 6942, 4290, respectively), EGFR (cat. # 3777, 4267, respectively), and AKT (cat. # 3787, 9272, respectively), along with markers such as HSP90 (cat. # 4877), HSP70 (cat. # 4872), HSP60 (cat. # 12165), CDK6 (cat. # 13331), and α-tubulin (cat. # 2144) (as a loading control). All antibodies were obtained from Cell Signaling Technology (CST, Danvers, MA, USA) and diluted 1/1000 as recommended. HRP-conjugated secondary antibodies (goat anti-rabbit IgG; Jackson ImmunoResearch, West Grove, PA, USA) were used, and chemiluminescent signal was visualized using an ECL detection system, following the method outlined by Mruk and Cheng [66]. Imaging was performed with the ImageQuant LAS4000 platform (GE Healthcare). Densitometry analysis for immunoblotting data was performed using ImageJ software (Wayne Rasband). The protocol for densitometry was provided by The University of Queensland with the recommendations from the works [67].

#### 2.3.3 Kinase Inhibitory Assay

The assay was performed using EGFR, HER2 and HER4 kinases from the Kinase Selectivity Profiling System TK-1 (Promega Corporation, Madison, WI, USA) and ADP-Glo Kinase assay kit (Promega Corporation, Madison, WI, USA). The inhibitory effect of the compounds was determined in accordance with the manufacturer’s technical manual. Kinase activity was measured by quantifying the amount of ATP remaining in solution following a kinase reaction. Luminescence detection was performed on a Tecan Infinite M200 PRO (Tecan, Grödig, Austria). Enzymatic activity inhibition percentage caused by studied compounds was evaluated at the single concentration of one μM. Lapatinib was used as a positive control.

#### 2.3.4 Determination of the Synergistic Effect of the Studied Compounds in Therapeutic Combinations

HCC1954 cells were seeded in 24-well plates (Corning) at a density of 5 × 10^4^ cells/well. After 24 h of incubation, test compound **ATF-2** and the chemotherapeutic agents alone or in combinations based on the respective IC_50_ values from the cytotoxicity assay were added to the cells. The maximum concentration of each drug was calculated to provide 80% cell mortality rate when used alone. The remaining concentrations of the test compounds were obtained by serial 2-fold dilutions of the maximum concentration. For everolimus, for which reliable half-inhibitory concentration data are not available, a concentration of 10 μM was chosen as the maximum concentration. Cell viability was assessed colorimetrically 72 h after drug addition using the MTT assay as described above. The open-source program SynergyFinder+ (https://synergyfinder.org) was used to calculate the synergistic indices of the combined use of standard chemotherapeutic drugs with the test compound using the HSA model, which implies finding such concentrations at which the expected combined effect will exceed the effect of using the drugs separately. Based on the values obtained using the model, heat maps were created to assess the therapeutic significance of drug combinations. Red areas, in this case, indicate a synergistic effect in the selected concentration range, and its therapeutic significance increases proportionally to the growth of color intensity and numerical values.

## 3 Results

### 3.1 Design and Molecular Modeling

**Figure 1:**
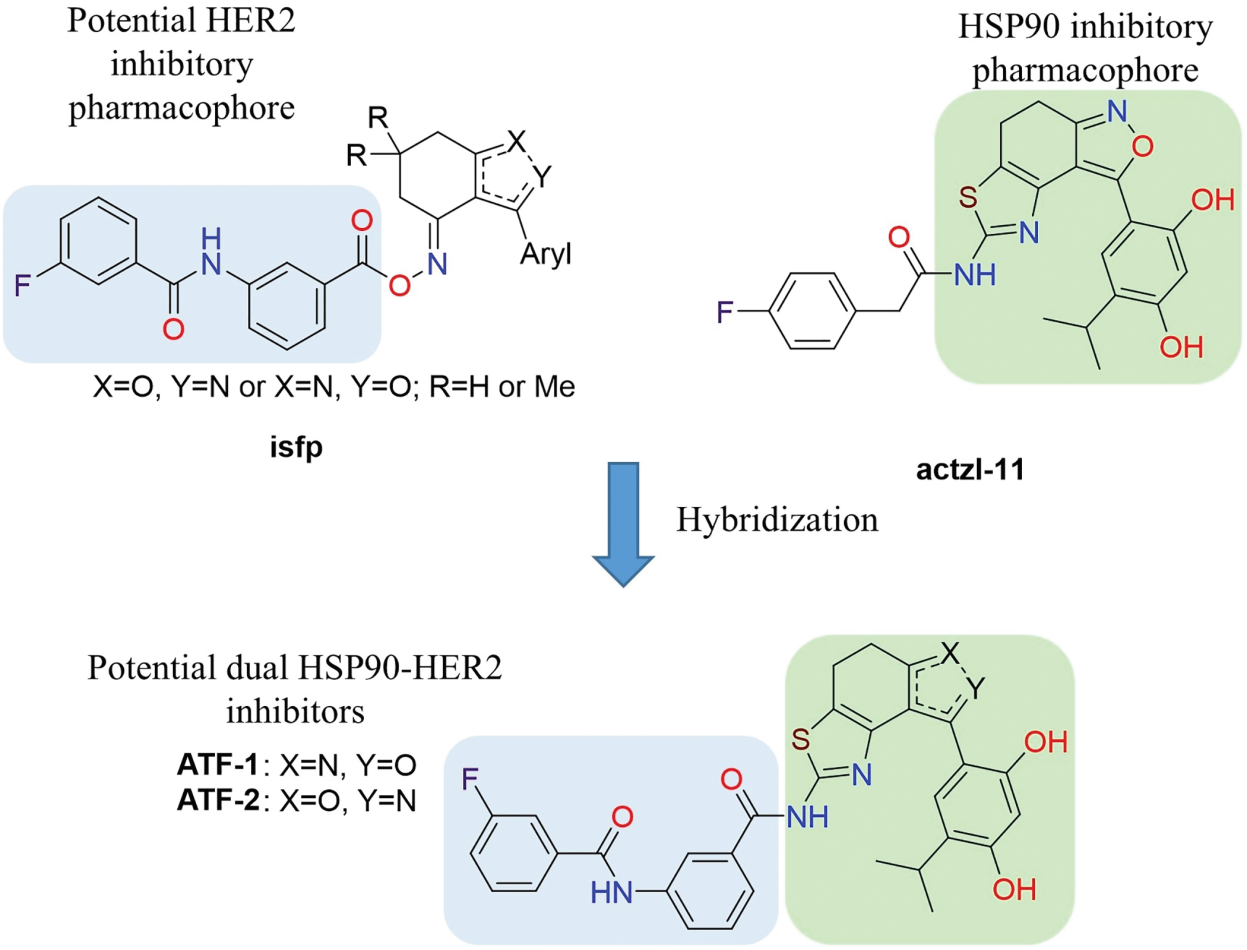
Design of potential dual HSP90-HER2 inhibitors

In the previous work dedicated to the search for new HSP90 inhibitors, we synthesized a series of *O*-acylated (*E*)-3-aryl-6,7-dihydrobenzisoxazol-4(*5H*)-one oximes **isfp** (Fig. 1). Some of which demonstrated moderate antiproliferative activity in breast cancer cells at low micromolar concentrations. All active compounds contained 3-(3-fluorobenzamido) benzoate as a common fragment. For a lead compound with Aryl = 4-hydroxy-3-methoxyphenyl, which is known as an HSP90 inhibitory pharmacophore [68]. The study showed that the antiproliferative effect is mainly due to inhibition of HSP90. However, for compounds with Aryl = fluoroaryl, we had suggestions, which were based on the docking experiments, that activity may be largely due to inhibition of tyrosine kinases (TKs), and 3-(3-fluorobenzamido)benzoate may serve as a TK inhibitory pharmacophore.

Another our work [55] developed a synthetic scheme for the preparation of polycyclic compounds containing isoxazole and thiazole rings and applied it for the synthesis of potential HSP90 inhibitor **actzl-11** (Fig. 1), which demonstrated antiproliferative activity against MCF-7 cells with an IC_50_ value of 9.5 μM and had remarkable effects on HSP90 client proteins. Based on the data obtained in these two works and our suggestions, the idea arose that hybridization of the mentioned molecules could be a possible opportunity to obtain a dual HSP90-HER2 inhibitor.

The study proposed two isomeric compounds, **ATF-1** and **ATF-2** (Fig. 1), that can potentially inhibit both HSP90 and HER2. For testing the hypothesis, we docked these compounds in the ATP-binding pocket of the HSP90α NTD (PDB ID 6LTI) and the ATP-binding pocket of the HER2 kinase domain (PDB ID 3PP0), carried out a molecular 200 ns dynamics simulation of obtained complexes followed by calculation of binding enthalpies with MM/GBSA method. Analogous calculations were made for the known HSP90 inhibitor luminespib and HER2 inhibitor lapatinib. As a result, we obtained ΔH_200_ values given in Table 1. In the case of HSP90, the predicted affinities of **ATF-1** and **ATF-2** were close and a little higher than the predicted affinity of luminespib. In the case of HER2, we obtained unexpected results, indicating that **ATF-2** has a high predicted affinity comparable to that of lapatinib, whereas the predicted affinity for **ATF-1** was much lower. For a double check of these results, we carried out 1000 ns simulations and calculated ΔH_1000_ MM/GBSA values based on these simulations and obtained almost the same results (Table 1). Additionally, for 1000 ns simulations, decomposition analysis (Table 2) was conducted.

**Table 1:** MM/GBSA ΔH values of binding of **ATF-1**, **ATF-2** and known inhibitors to HSP90α NTD and HER2 kinase domain

Compound	HSP90 (6LTI) MM/GBSA ΔH_**200**_, kcal/mol	HER2 (3PP0) MM/GBSA ΔH_**200**_, kcal/mol	HSP90 (6LTI) MM/GBSA ΔH_**1000**_, kcal/mol	HER2 (3PP0) MM/GBSA ΔH_**1000**_, kcal/mol
**Lapatinib**	–	−63.25	–	–
**Luminespib**	−52.66	–	–	–
**ATF-1**	−53.82	−46.62	−55.60	−45.55
**ATF-2**	−55.85	−59.99	−56.51	−62.22

Note: MM/GBSA, molecular mechanics/generalized born surface area; ATF-1, potential HSP90-HER2 dual inhibitor; ATF-2, potential HSP90-HER2 dual inhibitor; HSP, heat shock protein; HER2, human epidermal growth factor receptor 2; EGFR, epidermal growth factor receptor.

**Table 2:** Decomposition analysis showing the contributions of HER2 and HSP90α residues to MM/GBSA ΔH values for the formation of complexes with **ATF-1 and ATF-2**

HER2 Residues	ATF-1 ΔH, kcal/mol	ATF-2 ΔH, kcal/mol	HSP90α Residues	ATF-1 ΔH, kcal/mol	ATF-2 ΔH, kcal/mol
**LEU726**	−2.04	−1.75	LEU48	−0.70	−0.66
**VAL734**	−2.21	−2.64	ASN51	−4.30	−5.54
**ALA751**	−1.10	−1.28	SER52		−0.56
**ILE752**	−0.63	−0.82	ALA55	−1.46	−1.15
**LYS753**	−1.98	−4.10	ASP93	−0.59	
**ALA771**	−0.55	−0.69	ILE96	−1.25	−1.22
**MET774**	−0.51		GLY97	−1.35	−1.22
**LEU785**		−1.37	MET98	−2.96	−2.58
**LEU796**	−1.38	−1.86	LEU107	−1.46	−1.22
**THR798**	−1.10	−1.00	GLY108	−1.04	
*LEU800*	−1.11	−0.89	THR109	−1.39	
**MET801**		−0.53	GLY132		−1.00
**GLY804**		−0.91	GLN133	−0.55	−1.11
**CYS805**		−1.07	GLY135	−1.27	−1.28
**LEU852**	−0.97	−2.37	PHE138	−1.26	−1.42
**THR862**	−0.51	−2.02	VAL150	−0.61	−0.60
**ASP863**	−0.95	−1.29	THR184	−1.25	−1.45
**PHE864**	−1.19	−1.87	VAL186	−0.67	−0.73
**Ligand**	−22.94	−29.60	Ligand	−28.35	−28.85

Note: MM/GBSA, molecular mechanics/generalized born surface area; ATF-1, potential HSP90-HER2 dual inhibitor; ATF-2, potential HSP90-HER2 dual inhibitor; HSP, heat shock protein; HER2, human epidermal growth factor receptor 2; ΔH, enthalpy change.

After visual inspection of the obtained trajectories of 1000 ns simulations of **ATF**-HER2 complexes and decomposition analysis (Table 2), the difference in ΔH values for **ATF-1** and **ATF-2** can be explained by the fact that **ATF-2** can form a hydrogen bond between the oxygen atom of the isoxazole ring and the CYS805 sidechain of the protein. At the same time, polar contact between the nitrogen atom of the isoxazole ring of **ATF-1** and CYS805 is not stable.

**Figure 2:**
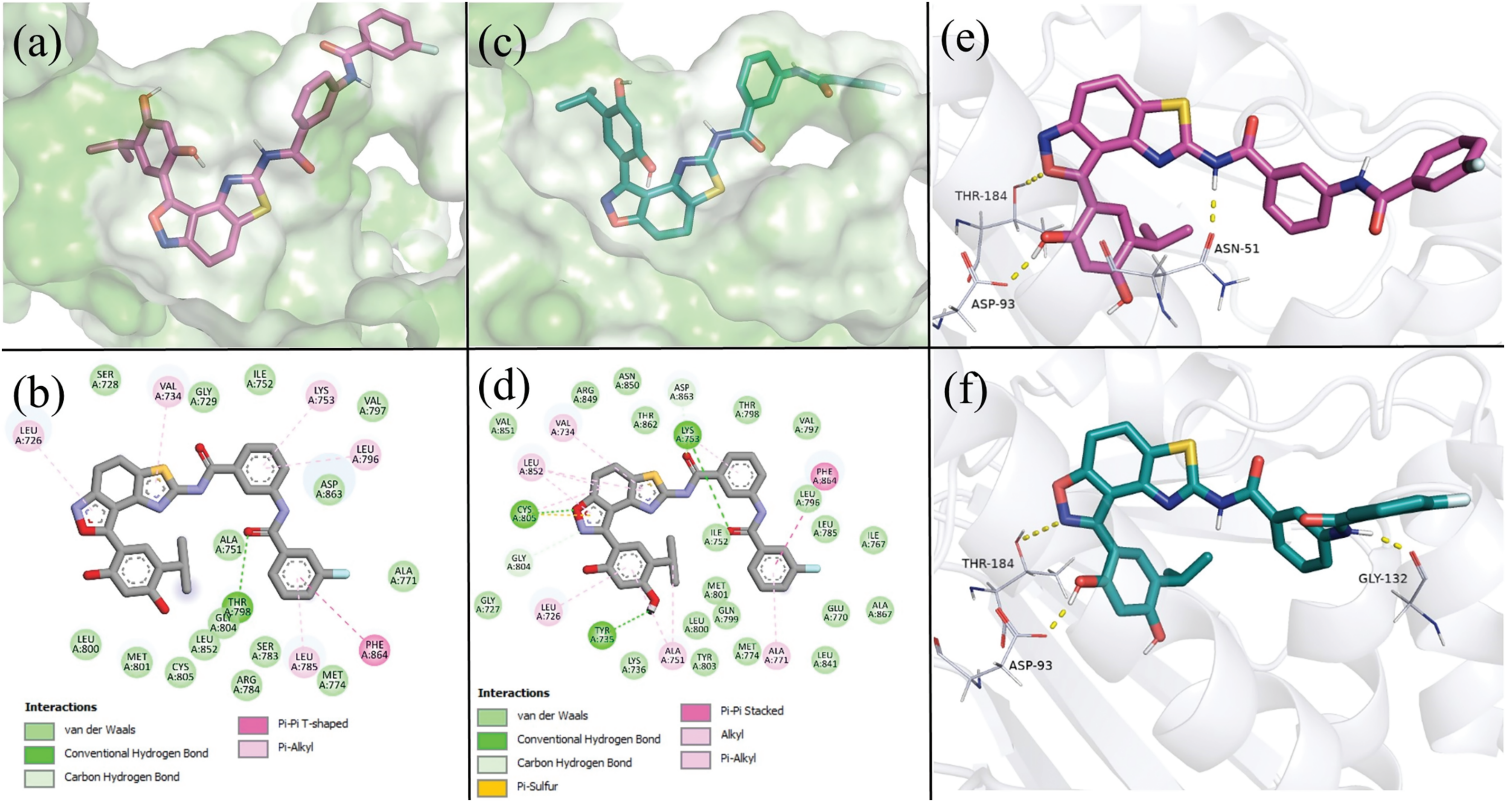
Predicted binding modes of **ATF-1** and **ATF-2** to HER2 and HSP90α, taken from a 1000 ns molecular dynamics simulation at t = 500 ns. (**a**) 3D representation of the predicted positions of **ATF-1** in the HER2 ligand-binding pocket. (**b**) 2D representation of intermolecular bonds of **ATF-1** with HER2. (**c**) 3D representation of the predicted positions of **ATF-2** in the HER2 ligand-binding pocket. (**d**) 2D representation of intermolecular bonds of **ATF-2** with HER2. (**e**) 3D representation of the binding mode and hydrogen bonds (shown as yellow dashed lines) of **ATF-1** with HSP90α. (**f**) 3D representation of the binding mode and hydrogen bonds (shown as yellow dashed lines) of **ATF-2** with HSP90α

Predicted binding modes of **ATF-1** (Fig. 2e) and **ATF-2** (Fig. 2f) to HSP90α are almost identical. Resorcinol-substituted isoxazole fragment fits in the same way as this fragment of luminespib, and 3-(3-fluorobenzamido) benzamide moiety can form hydrogen bonds with ASN51 and GLY132.

### 3.2 Chemistry

**Figure 3:**
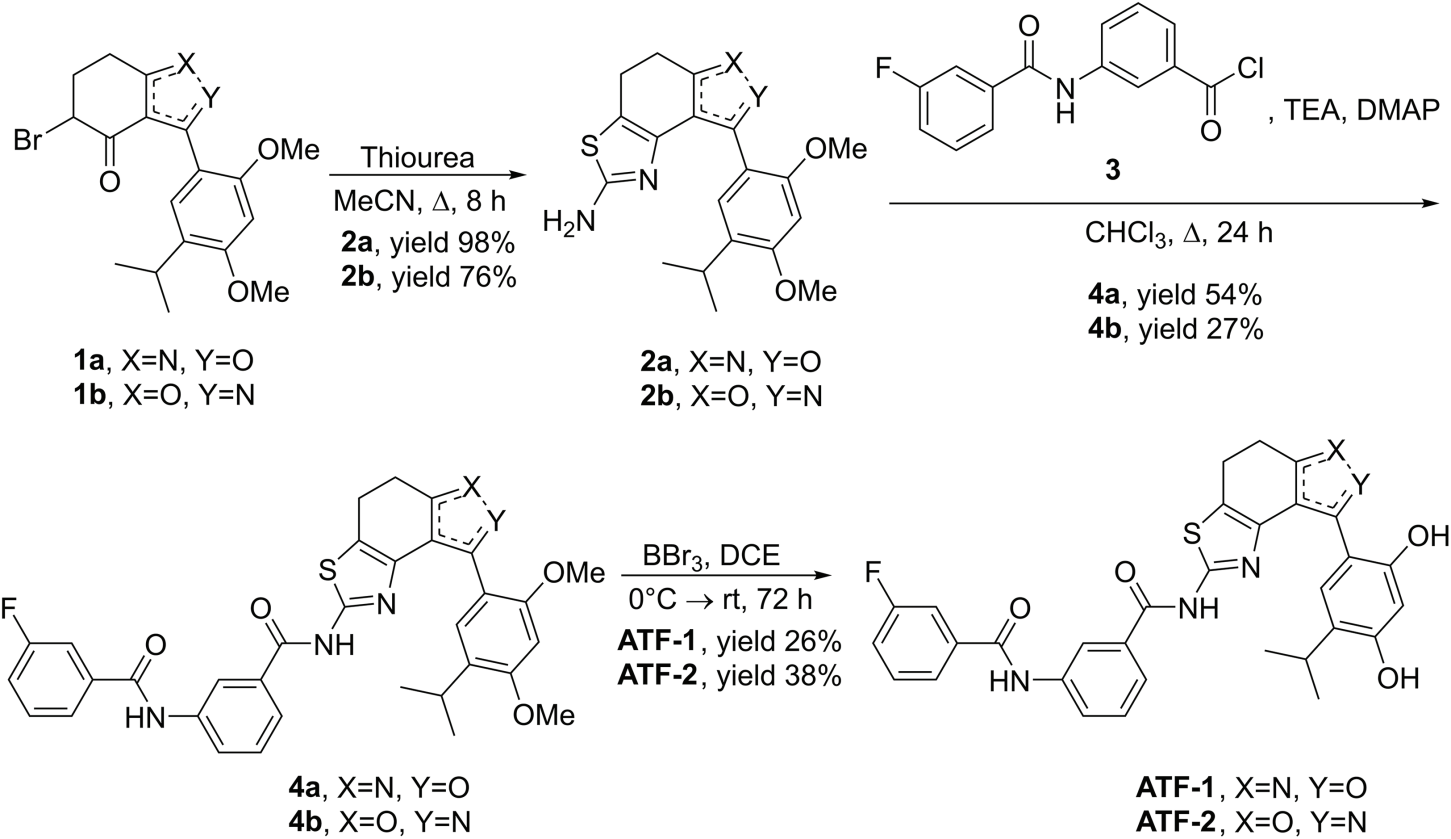
Synthesis of target compounds

For the synthesis of both target compounds, the study uses the approach that was developed earlier in our research group [55]. At the first stage, regioisomeric bromides **1** were condensed with thiourea in acetonitrile at reflux for 8 h. Under these conditions, aminothiazoles **2a** and **2b** were obtained in good yields, 98% and 76%, respectively. Then, acylation of aminothiazoles **2** with acyl chloride **3** was carried out in the presence of triethylamine and DMAP in CHCl_3_. Products **4a** and **4b** were obtained in yields of 54% and 27%, respectively. At the last stage, the methyl protecting groups were removed from the compounds **4a** and **4b** using excess BBr_3_ as a Lewis acid in dry dichloroethane (DCE), which gave the target compounds **ATF-1** and **ATF-2** in yields of 26% and 38%, respectively (Fig. 3).

### 3.3 Antiproliferative Activity and Selectivity of Synthesized Compounds

To study the antiproliferative activity of the synthesized hybrid compounds, presumably possessing dual inhibitory activity against both heat shock protein (HSP90) and human epidermal growth factor receptor type 2 (HER2), the HCC1954 breast cancer cell line was selected. Previously, we confirmed the high level of HER2 expression in these cells [69]. The known receptor tyrosine kinase inhibitor lapatinib was used as a reference drug. Based on the data obtained (Table 3), it can be concluded that the **ATF-1** and **ATF-2** molecules have antiproliferative activity in the micromolar concentration range. The values of half-inhibitory concentrations (IC_50_) were below 3 μM and were comparable to lapatinib.

**Table 3:** Antiproliferative activity of the synthesized compounds **ATF-1** and **ATF-2** and reference agents against breast cancer cells (n = 3)

**Compound**	**IC**_**50**_ **Values, Mean ± SD**, **μM**		**Selectivity index**
	HCC1954 (HER2+ Breast Cancer Cell Line)	hFB-hTERT (Normal Cells)	
**ATF-1**	1.69 ± 0.28	17.47 ± 1.53	10
**ATF-2**	2.07 ± 0.37	5.52 ± 0.54	3
**Lapatinib**	2.42 ± 0.30	12.80 ± 1.41	5
**Luminespib**	0.20 ± 0.01	0.080 ± 0.009	0.4

Note: HER2, human epidermal growth factor receptor 2; ATF-1, potential HSP90-HER2 dual inhibitor; ATF-2, potential HSP90-HER2 dual inhibitor.

A crucial stage in the investigation of novel medicinal compounds is the evaluation of their selectivity. Normal cell lines are used in these initial studies, which precede preclinical research. This assessment approach enables the preliminary evaluation of a new molecule’s safety profile, thereby reducing the likelihood of testing potentially toxic compounds on animals. In this study, telomerized fibroblasts (hFB-hTERT) were selected as models of normal cells.

One key parameter in rational drug design is the SI, defined as the ratio of a drug candidate’s IC_50_ value for a normal cell line to its IC_50_ value for a disease model cell line. A high SI value suggests that the investigated compound is potentially non-toxic and may be suitable for further *in vivo* testing. It is important to note that luminespib showed fibroblast toxicity, and the SI value for it was very low. The compounds **ATF-1** and **ATF-2** outperform luminespib by this criterion.

The obtained results indicate that the structural isomer **ATF-1** exhibits the highest selectivity toward tumor cells. Its IC_50_ for non-tumor cells is ten times higher than that for breast cancer cells. Notably, the SI of **ATF-1** surpasses that of lapatinib by a factor of two, highlighting its potential for the development of targeted therapies with reduced side effects and improved safety profiles. Compound **ATF-2** demonstrated lower selectivity compared to **ATF-1**.

### 3.4 Immunoblotting Analysis

**Figure 4:**
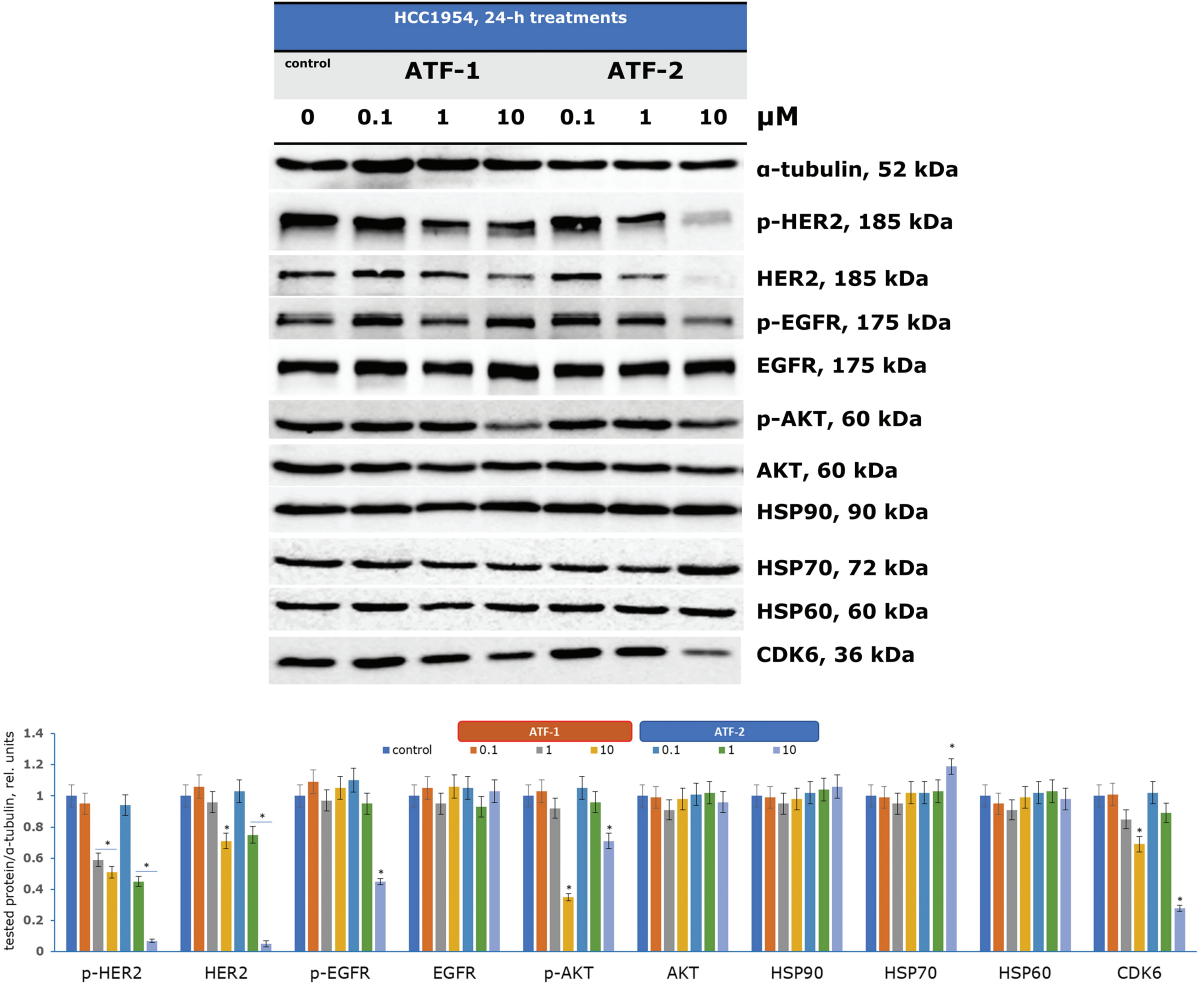
Regulation of signaling pathways in HCC1954 cells by dual HSP90-HER2 inhibitors. *p < 0.05 vs. control. The average value for the control was taken as one relative unit. Uncropped immunoblots are presented in Fig. S1 (ESI)

The next phase of the biological investigation involved evaluating changes in the expression of key proteins that regulate the growth and progression of HER2+ breast cancer cells (HCC1954) following a 24-h incubation with the two test compounds. Immunoblotting was employed to analyze alterations in signaling pathways (Fig. 4), with α-tubulin serving as a loading control.

Analyzing proteins associated with HSP90 enables the assessment of new compounds as potential dual inhibitors. Pharmacological inhibition of HSP90 is known to reorganize the entire chaperone signaling network [70,71]. Although neither **ATF-1** nor **ATF-2** directly affected HSP90 expression, a significant increase in HSP70 expression was observed upon treatment with 10 μM **ATF-2**, serving as a marker of HSP90 inhibition. Previous studies have similarly reported elevated HSP70 levels in various cells treated with investigational HSP90 inhibitors [72,73].

The HSP90 chaperone complex regulates the stability, activation, and maturation of over 400 client proteins, including oncogenic receptor tyrosine kinases (HER2, EGFR, MET), signaling proteins (AKT, ERKs), and cell cycle regulators [74]. Treatment with increasing concentrations of **ATF-1** led to a reduction in HER2 and its phosphorylated form (p-HER2) at 10 μM. **ATF-2** exhibited a stronger effect, decreasing HER2 and p-HER2 expression at just 1 μM, with significant suppression observed at 10 μM. Additionally, **ATF-2** inhibited another receptor tyrosine kinase, EGFR, and its activated form (p-EGFR) at 10 μM. These findings suggest that **ATF-2** has greater selectivity for HER2-dependent HCC1954 cells compared to **ATF-1**.

The antiproliferative effects of the synthesized compounds were confirmed by a reduction in phosphorylated AKT (p-AKT) expression at 10 μM, indicating an impact on the PI3K/AKT/mTOR signaling pathway, which is crucial for cell cycle regulation and tumor progression. Another key HSP90 client protein, cyclin-dependent kinase 6 (CDK6), plays a vital role in G1-phase cell cycle activation. Recent studies have also linked CDK6 overexpression to metabolic disruptions in cancer cells via the pentose phosphate pathway [75]. Following incubation with **ATF-1** and **ATF-2**, CDK6 expression was reduced at 10 μM.

### 3.5 Evaluation of the Inhibitory Effect of ATF-2 on ErbB Family Kinases

To further verify the potential of the compound **ATF-2** to inhibit HER2, we evaluated **ATF-2** as an inhibitor of the kinase activity of three kinases in the ErbB family. The tests were conducted at a single dose (1 μM), with lapatinib used as the positive control. The results are presented in Table 4.

**Table 4:** Inhibitory effects of **ATF-2** and the reference compound lapatinib on three kinases of the ErbB family (n = 3)

Kinase	Inhibition at One μM, Mean ± SD, %	
	Lapatinib	ATF-2
**EGFR/ErbB**	75 ± 8	53 ± 6
**HER2/ErbB-2**	71 ± 7	64 ± 5
**HER4/ErbB-4**	93 ± 8	81 ± 6

Note: EGFR, epidermal growth factor receptor; HER2, human epidermal growth factor receptor 2; HER4, human epidermal growth factor receptor 4; ATF-2, potential HSP90-HER2 dual inhibitor.

As can be seen from the obtained data, this test confirmed the ability of **ATF-2** to inhibit HER2, as well as EGFR and HER4. The effects of the selected compound were comparable to those of lapatinib.

### 3.6 Development of Therapeutic Combinations

Chemotherapy remains a key strategy for cancer treatment, but its clinical efficacy is often limited by tumor cell resistance to the drugs prescribed, as well as by the serious side effects that accompany their use. In this regard, there is an urgent need to develop new, no less effective approaches. One of the promising strategies that has received wide attention over the past few years is the development of synergistic combinations of targeted and chemotherapeutic drugs already used in clinical practice, as well as those that are at the stage of development and clinical trials [76–78].

As part of assessing the efficacy of using synthesized dual HER2-HSP90 inhibitors in combination therapy for HER2-positive breast cancer, therapeutic pairs consisting of the test compound **ATF-2** and standard chemotherapeutic drugs in different concentrations were developed and tested *in vitro*.

**Figure 5:**
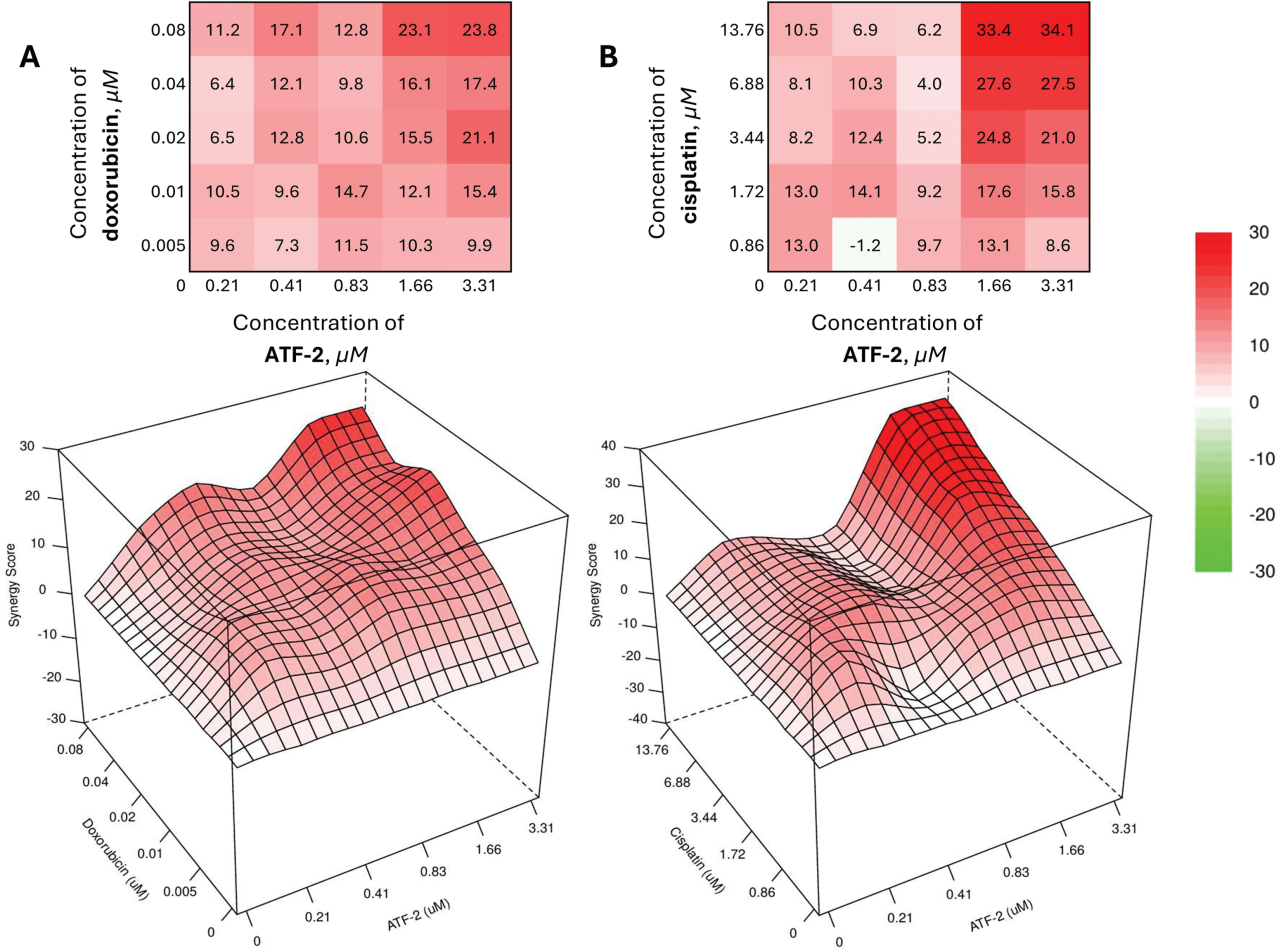
Combined effect of the synthesized compound **ATF-2** with doxorubicin (**A**) and cisplatin (**B**) on the HCC1954 cell line

The most successful combinations were **ATF-2** with doxorubicin (Fig. 5A), included in current clinical recommendations as part of the AC regimen, and cisplatin (Fig. 5B), used for the treatment of HER2-positive recurrent and metastatic breast cancer.

As can be seen from the data obtained using the open web resource SynergyFinder+ 3.0 [79,80], the best values of the synergy score (SS) calculated using the HSA model [81] are achieved with the combined use of chemotherapeutic drugs and **ATF-2** at concentrations that provide 40% cell death in a monotherapeutic regimen (0.04 + 1.66 μM for a combination with doxorubicin; 6.88 + 1.66 μM for a combination with cisplatin). Despite this, the greatest value in the development of effective therapeutic combinations is those pairs of drugs whose concentrations, when used together, are minimal. Upon analyzing the heat maps and 3D visualizations of the SS value matrix for doxorubicin and **ATF-2** combinations, as well as cisplatin and **ATF-2**, many regions emerge as particularly appealing in this context. Thus, a reliable synergistic effect (SS > 10) is achieved with the combined use of 0.41 μM **ATF-2** with 0.02 μM doxorubicin, while the effect can be slightly increased in the case of the reverse combination, in which 0.01 μM doxorubicin is used together with 0.83 μM of the test compound. In the case of combinations with cisplatin, two areas of the greatest synergistic effect can be identified, with one of them located in the zone of the lowest concentrations of both drugs (1.72 + 0.41 μM, SS = 14.1). The other area reflects the combined use of cisplatin at a concentration of 1.72 μM and **ATF-2** at a concentration that causes 40% death of HCC1954 cells when used individually (1.72 + 1.66 μM, SS = 17.6). In case of increasing the concentration of cisplatin by 2 times (3.44 μM), it is possible to achieve an increase in the synergistic effect by 1.4 times (SS = 24.8).

**Figure 6:**
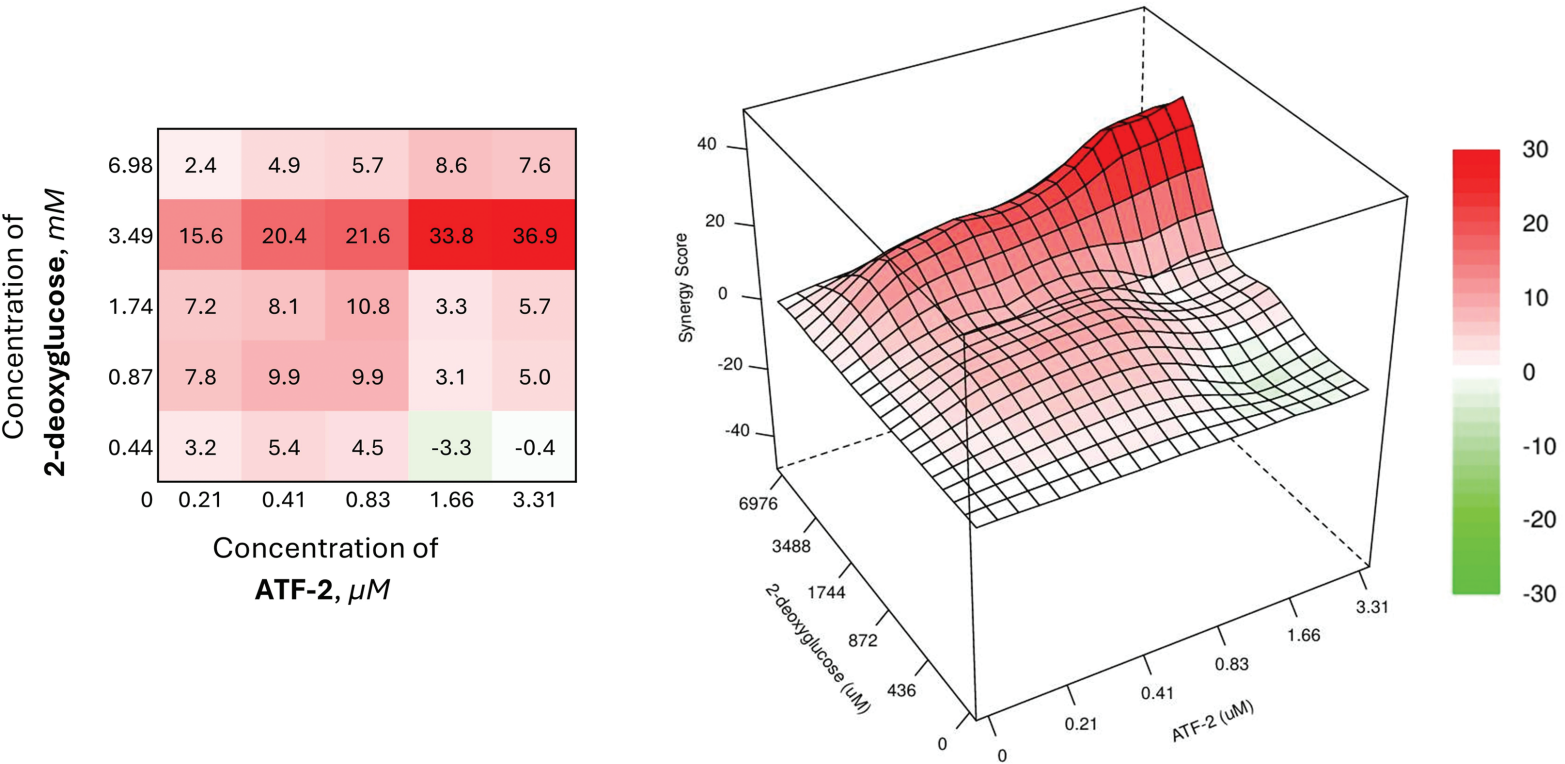
Combined effect of the synthesized compound **ATF-2** with 2-deoxyglucose on the HCC1954 cell line

Another example of an effective synergistic combination can be the joint use of the compound **ATF-2** with 2-deoxyglucose (Fig. 6).

The combined use of the drug at a concentration of 3.49 mM and the studied dual HER2-HSP90 inhibitor at its lowest concentration, causing 5% cell death, provides a synergistic effect with an SS value of 15.6. Increasing only the concentration of **ATF-2** to 0.41 and 0.83 μM, it is possible to enhance the synergistic effect of the combination (SS = 20.4 and 21.6, respectively). In the case of increasing the concentration of **ATF-2** to a value providing 40% cell death when used individually, the effectiveness of the combination increases sharply, causing a significant increase in the synergism index (SS = 33.8).

**Figure 7:**
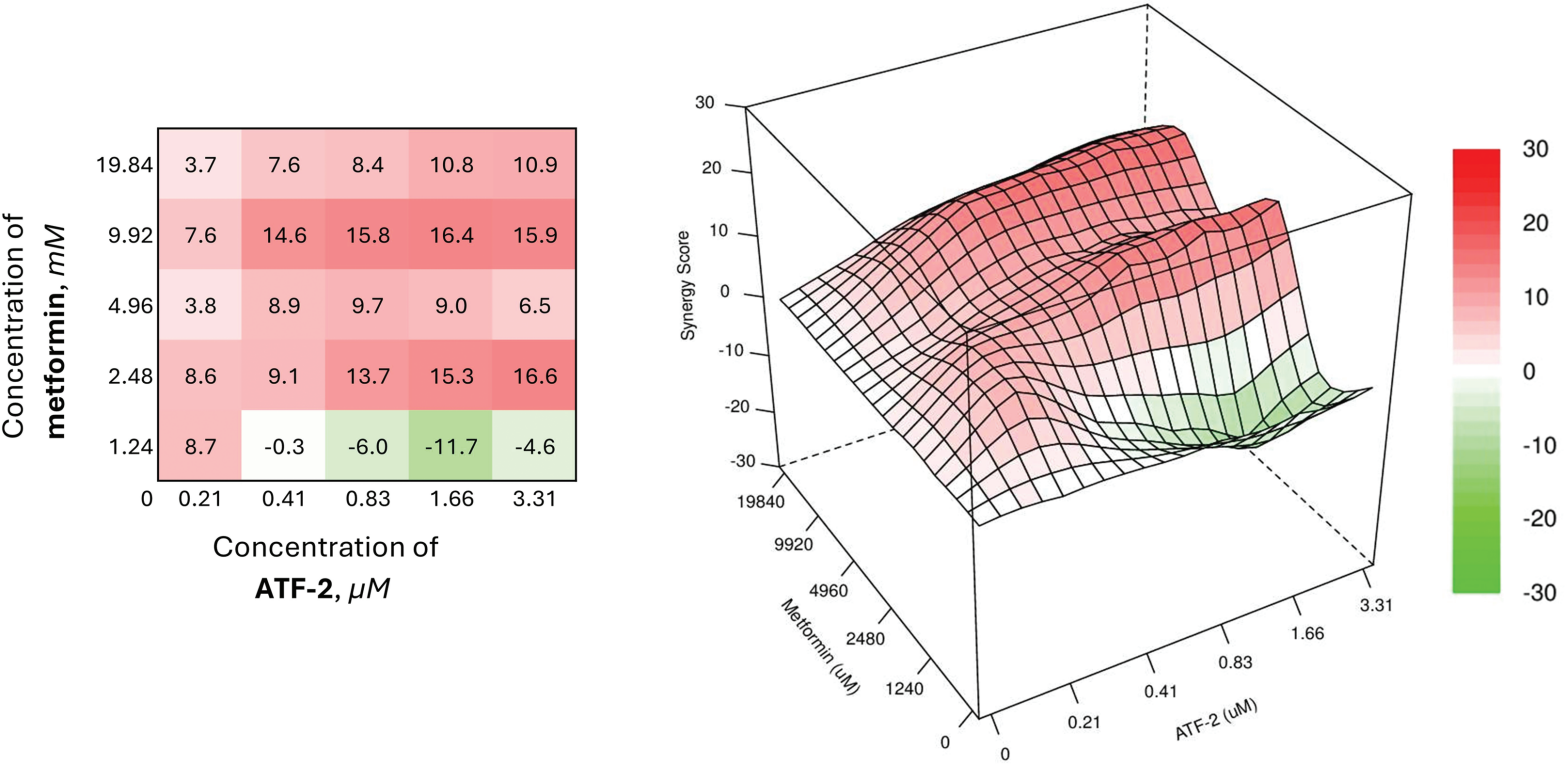
Combined effect of the synthesized compound **ATF-2** with metformin on the HCC1954 cell line

The therapeutic pairs of the studied compound with metformin have a similar activity profile (Fig. 7), demonstrating SS values similar to those for the combination of **ATF-2** at the lowest concentration and 2-deoxyglucose at a concentration of 3.49 mM.

The heat map of the combined use of a biguanide drug and the **ATF-2** molecule indicates the need to use the latter in a concentration range of 0.41 μM in the case of a combination with metformin at a concentration of 9.92 mM, or in a range of 0.83 μM when used together with metformin at 2.48 mM to achieve the desired synergistic effect. The best synergy score is achieved in the case of using 9.92 mM metformin and 1.66 μM **ATF-2** (SS = 16.4).

**Figure 8:**
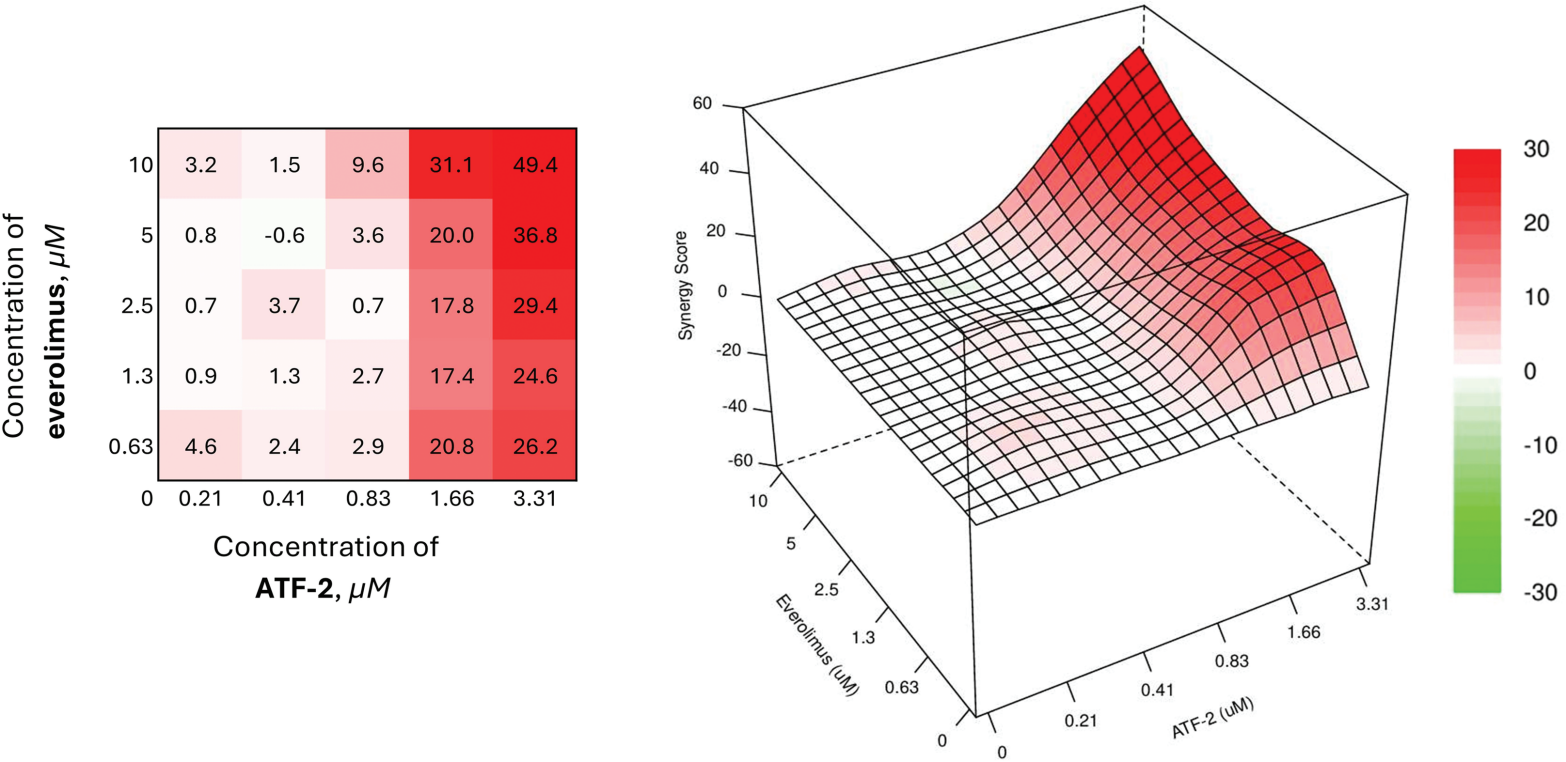
Combined effect of the synthesized compound **ATF-2** with everolimus on the HCC1954 cell line

Convincing results reflecting the synergistic effect of the chemotherapeutic combination are observed with the combined use of **ATF-2** and everolimus, a selective inhibitor of the serine-threonine kinase mTOR (Fig. 8).

The best results were achieved with a combination of 0.63 μM everolimus and 1.66 μM of the studied derivative (SS = 20.8). It is interesting to note that a similar value of the synergistic effect index is demonstrated by a combination in which the concentration of everolimus is increased almost 8 times. In addition, in the case of using the studied **ATF-2** molecule at a concentration of 1.66 μM with everolimus at concentrations of 1.3 and 2.5 μM, converging synergism scopes are also achieved — 17.4 and 17.8, respectively.

**Figure 9:**
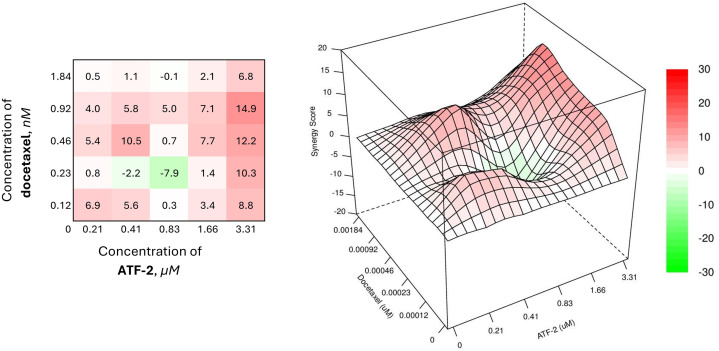
Combined effect of the synthesized compound **ATF-2** with docetaxel on the HCC1954 cell line

Combinations of the synthesized compound with docetaxel, a drug from the taxane group included in many adjuvant chemotherapy regimens for oncological diseases, were less effective against the HER2+ breast cancer cell model, and the SS values for such combinations were less than 10 (Fig. 9).

## 4 Discussion

HSP90 has been actively investigated over the past two decades as a promising molecular target in the development of new cancer therapies [82]. Currently, active research is ongoing, both into the discovery, evaluation, and application of HSP90 inhibitors [83–85], and into the potential of combining these inhibitors with other drugs to treat various types of cancer [86]. However, to date, only one HSP90 inhibitor has entered clinical practice. In Japan, the drug pimitespib was approved in 2022 for the treatment of gastrointestinal stromal tumors [87]. In our view, the limited success of HSP90 inhibitors can largely be attributed to the complexity of the HSP90 machinery, which involves interactions with various chaperones, co-chaperones, and other proteins [88,89]. The ATPase activity of HSP90 plays an important role in the function of this intricate system [90]. The primary strategy in developing anticancer agents targeting HSP90 focuses on designing binders to the ATP/ADP-binding site located in the N-terminal domain (NTD). Recombinant full-length HSP90 or its recombinant NTD are commonly used as models in such studies; however, in our opinion, these models do not adequately reflect the functioning of HSP90 in living cells. Consequently, selecting compounds for subsequent drug discovery phases based solely on their affinity to the recombinant chaperone is highly questionable. It is possible that a better approach to targeting cancer via HSP90 involves modulation rather than inhibition. In this context, cancer cells could serve as a model both for screening and for assessing the effects of active compounds on signaling pathways.

In recent years, growing attention has been drawn to strategies using HSP90 ligands in composite constructs. In particular, a number of studies have developed and evaluated PROTACs targeting HSP90 for degradation as antitumor agents [91–93]. One study [94] demonstrated the possibility of using hybrid molecules (HEMTACs)—containing both an HSP90 ligand and a ligand for a target protein—as a strategy to degrade that target. There’s also strong interest in exploring the potential of dual inhibitors targeting HSP90 as new anticancer therapies [95]. However, to the best of our knowledge, no HSP90-HER2 dual inhibitors have been described to date.

This work reports our efforts to design, synthesize, and evaluate HSP90-HER2 dual inhibitors. Using computer-aided design, we proposed two regioisomeric compounds, **ATF-1** and **ATF-2**, that can serve as ligands for both HSP90 and HER2. Based on molecular docking, molecular dynamics simulations, and MM/GBSA calculations, compound **ATF-2** demonstrated potential to bind both target proteins with affinities comparable to those of the well-known HSP90 and HER2 inhibitors, luminespib and lapatinib, respectively. The second compound, **ATF-1**, based on the same calculations, appears less promising as a HER2 ligand but still shows potential as an HSP90 ligand. Both designed molecules were synthesized from two regioisomeric 4,5-dihydrothiazolo [5^′^,4^′^:5,6]benzisoxazol-2-amines **2a**-**b** and 3-(3-fluorobenzamido)benzoic acid as key building blocks.

Methods for analyzing HSP90 inhibitors encompass a wide range of approaches, from molecular biology to proteomics and high-throughput biophysical assays [33,96,97]. A key indicator of HSP90 inhibitor efficacy is the degradation of client proteins [98], which can be reliably measured using immunoblotting, mass spectrometry, fluorescence-based assays, and functional tests. The integration of these methods is crucial for understanding the mechanism of action and optimizing HSP90 inhibitors as anticancer agents. For the *in vitro* phase of the study, aggressive HER2-positive breast cancer cells were used. **ATF-2** exhibited significant antiproliferative effects, mediated through its influence on HER2 signaling. The compound modulated both the phosphorylated and total forms of the HER2 protein, suggesting a complex mechanism of action. Additionally, kinase inhibition assays revealed that at one μM, compound **ATF-2** inhibited three kinases of the ErbB family: EGFR by 53%, HER2 by 64%, and HER4 by 81%.

Extended biological testing suggests the molecule’s high potential for use in chemotherapeutic combinations. Specifically, **ATF-2** demonstrated efficacy in combination with doxorubicin, cisplatin, 2-deoxyglucose, and metformin. This finding opens the possibility of reducing chemotherapy dosages to minimize overall treatment toxicity.

Thus, after analyzing the data from molecular modeling and *in vitro* experiments, it can be concluded that the proposed structures are of interest as dual inhibitors of HSP90 and tyrosine kinases. In particular, these findings highlight the potential of **ATF-2** as a dual HSP90-HER2 inhibitor, exhibiting broader inhibitory effects on the HER2, EGFR, and CDK6 pathways.

A key limitation of this study is the small number of compounds tested, which consequently prevents meaningful structure–activity relationship (SAR) analysis. Further work in this direction may include the replacement of 3-(3-fluorobenzamido)benzamide, used in this research as the HER2 binding fragment, with another fragment that would fit in the ATP-binding pocket of different tyrosine kinases. This strategy can lead to the development of more effective and selective HER2-HSP90 and other tyrosine kinase–HSP90 dual inhibitors.

## 5 Conclusion

Thus, after analyzing the data from molecular modeling and *in vitro* experiments, it can be concluded that the proposed structures are of interest as dual inhibitors of HSP90 and tyrosine kinases. In particular, these findings highlight the potential of **ATF-2** as a dual HSP90-HER2 inhibitor, exhibiting broader inhibitory effects on the HER2, EGFR, and CDK6 pathways. Further work in this direction may include the replacement of 3-(3-fluorobenzamido)benzamide, used in this research as the HER2 binding fragment, with another fragment that would fit in the ATF-binding pocket of different tyrosine kinases. This strategy can lead to the development of more effective and selective HER2-HSP90 and other tyrosine kinase–HSP90 dual inhibitors.

## Supplementary Materials



## Data Availability

Data available within the article or its supplementary materials.

## Supplementary Materials

Electronic supplementary information (ESI) comprises NMR data (^1^H, ^13^C, ^19^F), HRMS data, HPLC data and uncropped immunoblots. Supplementary data to this article can be found online at https://www.techscience.com/doi/10.32604/or.2025.067832/s1.
