# Supplementary material for: Targeting HER2-Positive HCC1954 Breast Cancer Cells by Novel Thiazole-Dihydrobenzisoxazoles: In-Depth Design, Synthesis and Initial In Vitro Study
Source: Oncol Res. 2025 Nov 27;33(12):4049–72. doi: 10.32604/or.2025.067832 (PMC12796772; doi:10.32604/or.2025.067832)
Supplement: Supplementary file 1 [file OncolRes-33-67832-s001.docx]

**SUPPORTING INFORMATION**

Targeting HER2-positive HCC1954 breast cancer cells by novel thiazole-dihydrobenzisoxazoles: in-depth design, synthesis and initial in vitro study

Yuri A. Piven^1^, Danila V. Sorokin^2^, Nastassia A. Varabyeva^1^, Alexandra L. Mikhaylova^2^, Fedor B. Bogdanov^2^, Elena V. Shafranovskaya^1^, Raman M. Puzanau^3^, Fedor A. Lakhvich^1^ and Alexander M. Scherbakov^2,4*^

^1^Institute of Bioorganic Chemistry, National Academy of Sciences of Belarus, Minsk 220084, Belarus

^2^Department of Experimental Tumor Biology, Blokhin N.N. National Medical Research Center of Oncology, Moscow, 115522, Russia

^3^Health Institution "National Anti-Doping Laboratory", Minsk, 223040, Belarus

^4^Gause Institute of New Antibiotics, Moscow, 119021, Russia

^*^Corresponding Author: Alexander Mikhailovich Scherbakov. Email: alex.scherbakov@gmail.com

Received: Day Month 2025; Accepted: Day Month Year; Published: Day Month Year

**ABSTRACT:**

**Objectives:** The study aimed to develop small-molecule potential dual HSP90-HER2 inhibitors and evaluate them as anticancer agents in HER2-positive cells.

**Methods:** The research project involved obtaining a series of compounds with potential dual inhibitory activity against HSP90 and HER2 by targeted organic synthesis, which was preliminarily assessed using molecular modelling and calculation of key parameters of molecular dynamics. The potential therapeutic benefit of the obtained molecules was studied using basic molecular biological methods, including assessment of cytotoxic activity *in vitro* using the MTT test, as well as determination of a possible mechanism of action based on the expression of key participants in intracellular signaling (western blotting). Additionally, therapeutic combinations were developed and tested on a cellular model of the disease, including a lead Rcompound and chemotherapeutic drugs used in clinical practice, in order to find synergistic pairs and improve the effectiveness of the treatment.

**Results:** In this work, novel dual HSP90-HER2 inhibitors, based on the fused thiazole-dihydrobenzisoxazole polycyclic scaffold, were designed and synthesized. The resulting compounds exhibited strong antiproliferative activity against HER2-positive breast cancer cells with high selectivity. Among them, **ATF-2** demonstrated antiproliferative activity comparable to HER2 inhibitor lapatinib and significantly suppressed HER2 expression and activity, epidermal growth factor receptor (EGFR) activity, and cyclin-dependent kinase 6 (CDK6) expression in HCC1954 breast cancer cells.

**Conclusion:** These findings highlight **ATF-2** as a promising dual HSP90-HER2 inhibitor with broader inhibitory effects on the HER2, EGFR, and CDK6 pathways.

**KEYWORDS:** anticancer therapy; breast cancer; heat shock protein 90 (HSP90); human epidermal growth factor receptor 2 (HER2); epidermal growth factor receptor (EGFR); dual inhibitors

**Table of contents**

^[1](#_Toc205572163)^[H](#_Toc205572163) [NMR,](#_Toc205572163) ^[13](#_Toc205572163)^[C NMR and](#_Toc205572163) ^[19](#_Toc205572163)^[F NMR Spectra 4](#_Toc205572163)

[HRMS Spectra 13](#_Toc205572164)

[HPLC of compounds ATF-1 and ATF-2 15](#_Toc205572165)

[Uncropped Immunoblots 16](#_Toc205572166)

**Ошибка! Недопустимый объект гиперссылки.Ошибка! Недопустимый объект гиперссылки.Ошибка! Недопустимый объект гиперссылки.**

## ^1^H NMR, ^13^C NMR and ^19^F NMR Spectra

^1^H NMR (500 MHz, DMSO-d6) of compound **2b**

^13^C NMR (126 MHz, DMSO-d6) of compound **2b**

^1^H NMR (500 MHz, DMSO-d6) of compound **4a**

^13^C NMR (126 MHz, DMSO-d6) of compound **4a**

^19^F NMR (470 MHz, DMSO-d6) of compound **4a**

^1^H NMR (500 MHz, DMSO-d6) of compound **4b**

^13^C NMR (126 MHz, DMSO-d6) of compound **4b**

^19^F NMR (470 MHz, DMSO-d6) of compound **4b**

^1^H NMR (500 MHz, DMSO-d6) of compound **ATF-1**

^13^C NMR (126 MHz, DMSO-d6) of compound **ATF-1**

^19^F NMR (470 MHz, DMSO-d6) of compound **ATF-1**

^1^H NMR (500 MHz, DMSO-d6) of compound **ATF-2**

^13^C NMR (126 MHz, DMSO-d6) of compound **ATF-2**

^19^F NMR (470 MHz, DMSO-d6) of compound **ATF-2**

## HRMS Spectra

HRMS of **2b**

HRMS of **4a**

HRMS of **4b**

HRMS of **ATF-1**

HRMS of **ATF-2**

## HPLC of compounds ATF-1 and ATF-2

| **Integration Peak List** | | |  |  |  |  |  |  |  |  |  |  |
| --- | --- | --- | --- | --- | --- | --- | --- | --- | --- | --- | --- | --- |
| **Peak** | **Start** | **RT** | **End** | **Height** | | | **Area** | | | **Area %** | | |
| 1 | 7,15 | 7,23 | 7,44 | 777,48 | | | 4291,66 | | | 100 | | |

HPLC of **ATF-1**

| **Integration Peak List** | | |  |  |  |  |  |  |  |  |  |  |
| --- | --- | --- | --- | --- | --- | --- | --- | --- | --- | --- | --- | --- |
| **Peak** | **Start** | **RT** | **End** | **Height** | | | **Area** | | | **Area %** | | |
| 1 | 7,09 | 7,2 | 7,44 | 499,09 | | | 2703,83 | | | 100 | | |

HPLC of **ATF-2**

## Uncropped Immunoblots


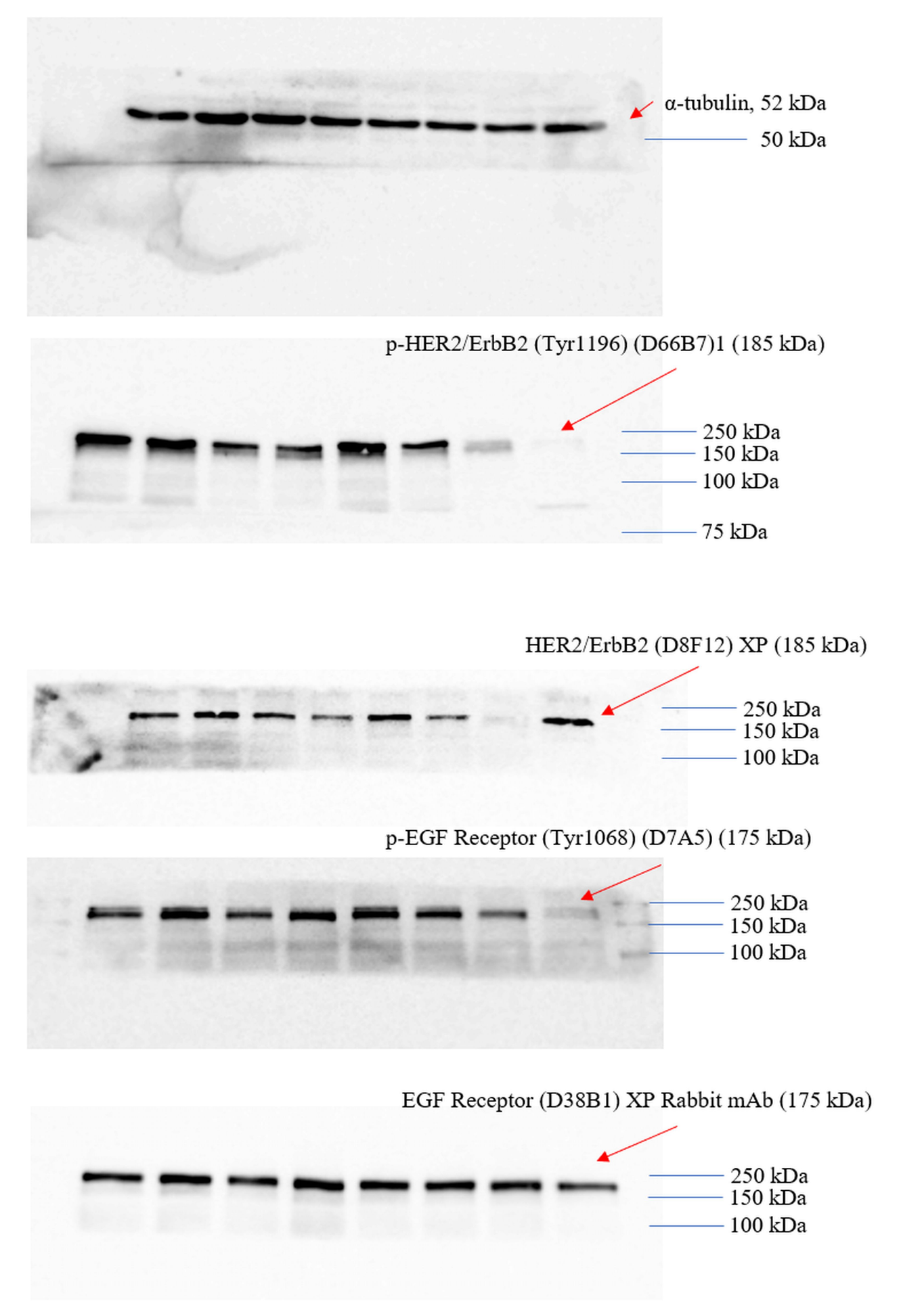


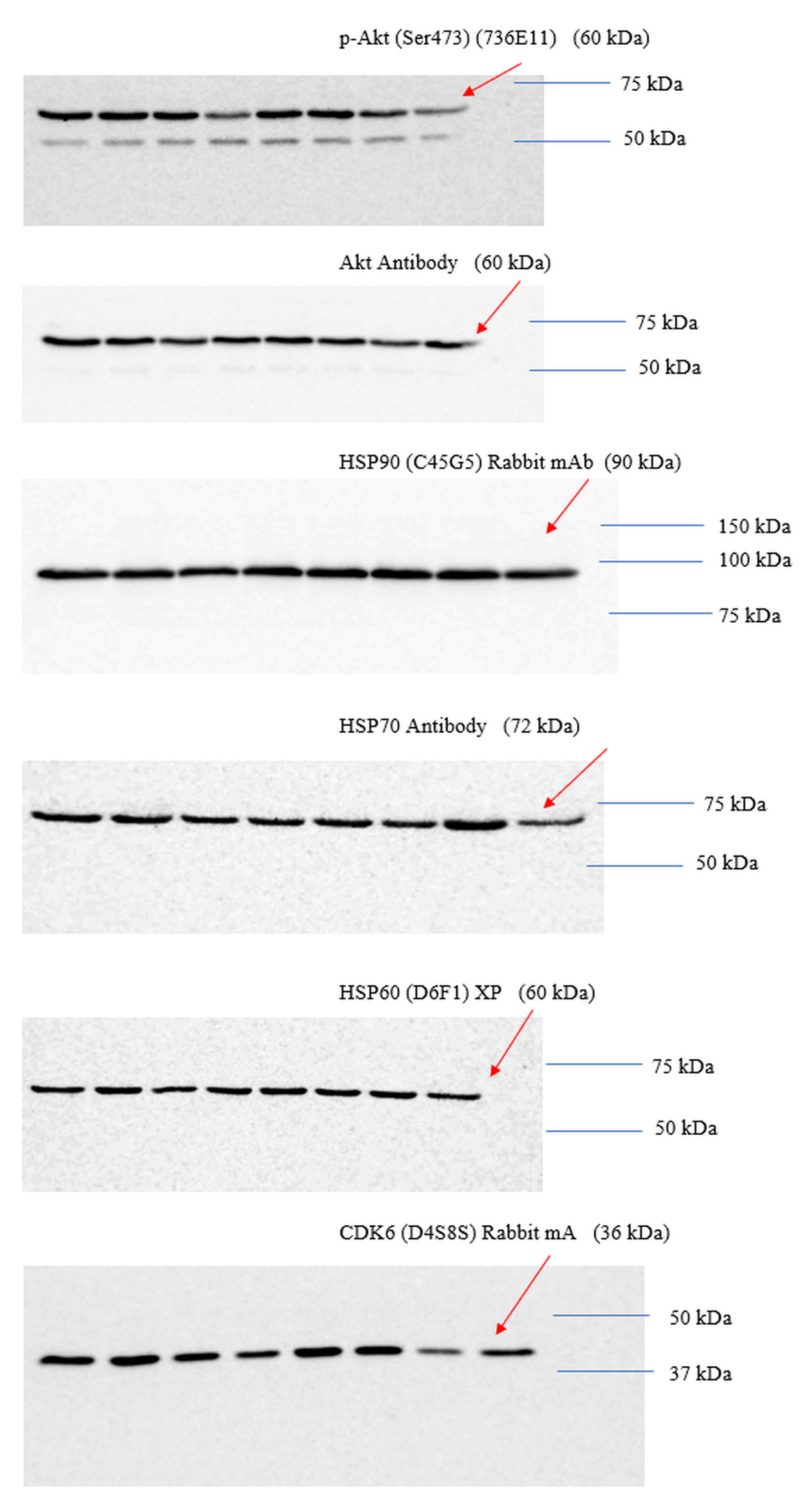


Figure S1. Uncropped immunoblots. On each blot, the first 7 tracks were cut out for Figure 4.
